# Introduction of a Dedicated Induction Programme for International Medical Graduates in Psychiatry Training at South London and Maudsley National Health Service (NHS) Foundation Trust and Oxleas NHS Foundation Trust

**DOI:** 10.1192/bjo.2025.10308

**Published:** 2025-06-20

**Authors:** Ajasra Sheokand, Rajakshi Khanna, Dianne Estrada, Shivani Dudha, Ruchika Rathee

**Affiliations:** South London and Maudsley NHS Trust, London, United Kingdom

## Abstract

**Aims:** International Medical Graduates (IMG) constitute a significant portion of the NHS workforce and play a vital role in psychiatric care. However, many face challenges in navigating the United Kingdom (UK) healthcare system, medico-legal responsibilities, and cultural differences, particularly when psychiatry training is their first NHS role. Despite these challenges, no dedicated IMG induction programme previously existed at South London and Maudsley (SLaM) and Oxleas NHS Foundation Trusts. Existing induction sessions were not tailored to IMG-specific needs, leaving trainees without structured guidance. Feedback from IMGs highlighted the need for a formal induction to provide essential training and signpost available support.

**Methods:** A structured International Medical Graduate Induction Programme was introduced as a mandatory, protected-time event for new core and higher psychiatry trainees. The first session, held in August 2024, was delivered by consultants, IMG core and speciality trainees, British Medical Association representatives, and medical indemnity advisors. The programme covered supervision and support in psychiatry training, medico-legal responsibilities, the Mental Health Act assessment, documentation standards, differential attainment, and practical aspects of living in London.

**Results:** Ten IMGs attended the August 2024 session, with six providing feedback. All respondents (100%) reported that the programme met their expectations. The most valued sessions were Mental Health Act assessment training and documentation in psychiatry. Feedback suggested a need for greater clarity on support services and professional indemnity.

**Conclusion:** The introduction of a structured IMG induction programme at SLaM and Oxleas NHS Foundation Trust was well received with positive feedback reinforcing its value in supporting IMG trainees’ transition into UK psychiatry training, enhancing their confidence and competency. The plan is to integrate this with routine inductions for each new cohort of trainees. The next IMG induction is in February 2025 for which more than 15 new IMG trainees have signed up. Attendance remains mandatory for new core and higher psychiatry trainees. Future sessions will incorporate feedback to further refine the programme, ensuring it remains relevant and responsive to the needs of IMG trainees.

